# Promoting health through sports–medicine integration in China: a narrative and conceptual review of challenges, international

**DOI:** 10.3389/fpubh.2026.1958935

**Published:** 2026-10-13

**Authors:** Yang Yang, Guo Jing

**Affiliations:** 1 Physical Education Department, China Agricultural University, Beijing, China; 2 Beijing Sport University, Beijing, China

**Keywords:** China, exercise prescription, health promotion, integrated care, narrative review, noncommunicable diseases, physical activity, sports–medicine integration

## Abstract

Physical inactivity and noncommunicable diseases (NCDs) pose sustained challenges to population health, while prevention, clinical care, rehabilitation, and community-based physical activity are often delivered through separate professional and administrative systems. In China, sports–medicine integration has been promoted as a strategy to connect national fitness, health promotion, chronic disease management, and rehabilitation. However, its conceptual boundaries, implementation mechanisms, evidence base, and potential risks require more critical examination. This article uses a narrative and conceptual review approach. This structured narrative and conceptual review used PubMed as the principal bibliographic database, supplemented by backward citation searching, targeted searches of authoritative governmental and professional sources, and relevant Chinese-language literature. Sources were screened according to predefined relevance criteria and critically appraised according to study design, methodological rigor, directness, recency, and source authority. Seminal conceptual sources were retained without a lower date limit, whereas evidence describing contemporary health systems and implementation was preferentially drawn from 2000–August 2026, with post-2020 literature prioritized where available. Peer-reviewed empirical studies, systematic/scoping reviews, and authoritative documents from national governments, the World Health Organization (WHO), and professional bodies were prioritized over commercial market reports, media commentary, and indirect citations. Evidence was synthesized thematically rather than quantitatively. The literature supports a stronger role for physical activity in health promotion and chronic disease prevention, but it does not justify treating sports–medicine integration as a self-evidently effective policy solution. China has important enabling conditions, including national policy support and growing exercise-prescription capacity, yet implementation remains constrained by administrative fragmentation, uneven local capacity, incomplete professional credentialing, financing uncertainty, and limited evaluation at scale. International experience illustrates complementary strengths: the United States has developed professional certification and health-system implementation models; Germany links medical recommendations with qualified community sport and insurance-based prevention; and Japan combines national physical activity guidance with health-promotion and community-care structures. Sports–medicine integration should be understood as a coordinated, risk-stratified health-service strategy rather than as the medicalization of all physical activity. Future development in China should emphasize clear governance, standardized referral pathways, recognized professional scopes of practice, sustainable financing, interoperable data systems, and explicit evaluation of effectiveness, cost-effectiveness, scalability, and equity.

## 1 Introduction

China is undergoing a major demographic and epidemiological transition characterized by population ageing, urbanization, changing lifestyles, and a high burden of chronic noncommunicable disease. NCDs dominate mortality in China, and physical inactivity is an important modifiable risk factor for cardiovascular disease, type 2 diabetes, several cancers, impaired functional capacity, and poorer mental health (1, 2). Globally, an estimated 31.3% of adults did not meet recommended levels of physical activity in 2022, and current trends indicate that the world is off track to achieve the 2030 target for reducing insufficient physical activity (3). These trends strengthen the case for prevention strategies that extend beyond episodic clinical treatment and address health behavior, living environments, and long-term self-management.

Physical activity is one of the few health behaviors with broad benefits across prevention, treatment support, rehabilitation, healthy ageing, and mental wellbeing. WHO recommends that adults accumulate 150–300 min of moderate-intensity aerobic physical activity, 75–150 min of vigorous-intensity activity, or an equivalent combination each week, while also limiting sedentary time (4, 5). The WHO Global Action Plan on Physical Activity further emphasizes that increasing physical activity is not solely an individual behavioral task; it requires coordinated action across health, sport, education, transport, urban planning, workplaces, communities, and civil society (6). Thus, the public-health value of physical activity is well established, but the organizational question of how physical activity should be connected to healthcare remains unresolved in many countries. China provides a particularly relevant context for examining this question. The Healthy China 2030 Plan calls for health promotion across the life course, integration of health into public policy, improvement of national fitness, and stronger coordination between prevention, treatment, and rehabilitation (7). The Law of the People’s Republic of China on Basic Medical and Health Care and the Promotion of Health also establishes health promotion, prevention, national fitness, and cross-sectoral responsibility within the legal framework of health governance (8). In practice, however, sports and healthcare services have historically developed through different administrative systems, professional pathways, information infrastructures, and financing arrangements. Recent analyses of sports–medicine integration in China identify persistent problems including the absence of a unified implementation model, limited exercise-prescription services in hospitals, weak professional certification, and concentration of early pilots in relatively well-resourced urban settings (9).

International experience suggests that no single governance model provides a complete solution. Decentralized and insurance-based systems may support professional specialization, flexible service provision, and private-sector or community innovation, while also creating fragmentation and unequal access. More centralized policy systems can align national priorities and mobilize resources, but may still face administrative silos, local implementation gaps, and uneven responsiveness to diverse populations. A balanced analysis therefore requires attention to both the enabling features and the limitations of different models rather than assuming the inherent superiority of either state-led or market-oriented arrangements.

Accordingly, this narrative and conceptual review critically examines sports–medicine integration as a health-promotion strategy, with a primary focus on China and selected comparisons with the United States, Germany, and Japan. The review has four objectives: (1) to clarify the conceptual rationale for linking physical activity and healthcare; (2) to examine governance, service-delivery, workforce, financing, equity, and information challenges in China; (3) to identify relevant strengths and limitations of selected international approaches; and (4) to propose priorities for a more effective, equitable, and sustainable model of sports–medicine integration. The review is intentionally China-centered rather than a comprehensive global comparison.

## 2 Methods

### 2.1 Review design and scope

This study was designed as a structured narrative and conceptual review rather than a systematic review or meta-analysis. The purpose was to synthesize conceptual, policy, health-system, and implementation evidence relevant to sports–medicine integration, with China as the primary focus and the United States, Germany, and Japan used as selected international comparators. The review therefore emphasized thematic interpretation, conceptual comparison, and critical appraisal of implementation issues rather than exhaustive identification of all eligible publication.

### 2.2 Information sources and search strategy

PubMed was used as the principal bibliographic database for English-language peer-reviewed literature. Searches were supplemented by citation chaining from relevant reviews and policy analyses, targeted searches of authoritative government and professional websites, and Chinese-language literature already used in the underlying manuscript where it remained relevant and sufficiently authoritative. Authoritative sources included publications from the World Health Organization, national health and sports authorities, statutory health agencies, and recognized professional organizations.

The search strategy combined terms relating to physical activity and exercise with terms relating to healthcare integration and service delivery. Search concepts included combinations of “sports medicine integration,” “exercise prescription,” “exercise referral,” “physical activity promotion,” “Exercise is Medicine,” “integrated care,” “primary care,” “rehabilitation,” “health promotion,” “Healthy China,” and country-specific terms for China, the United States, Germany, and Japan. Searches were updated through August 2026.

A representative PubMed search combined terms for physical activity or exercise-based intervention with terms for healthcare delivery and integration. The complete search strategy, including Boolean operators and country-specific adaptations, is provided in (Table 1). No strict lower publication-date limit was applied to seminal conceptual sources, such as Crawford’s original formulation of healthism. For contemporary health-system descriptions and implementation evidence, literature published from 2000 onward was emphasized, with post-2020 sources prioritized wherever they could replace older descriptions of current practice. Peer-reviewed original research, systematic reviews, scoping reviews, and official publications from WHO, national health ministries, statutory health bodies, and recognized professional organizations were prioritized. Commercial market reports, media commentary, unpublished theses, and secondary quotations were not used as the principal evidence for foundational theoretical claims, national statistics, or current international system descriptions when more authoritative sources were available.

**Table 1:** Literature search strategy.

**Component**	**Search terms**
Physical activity	“physical activity” OR “exercise prescription” OR “Exercise is Medicine”
Integration	“integrated care” OR “sports–medicine integration” OR “health promotion”
China	China OR Chinese OR “Healthy China”
USA	“United States” OR USA OR American
Germany	Germany OR German
Japan	Japan OR Japanese
Date	Updated to August 2026
Database	PubMed

### 2.3 Eligibility criteria and study selection

Sources were considered eligible when they addressed at least one of the following: (1) integration of physical activity or exercise services with healthcare; (2) exercise prescription, exercise referral, or rehabilitation pathways; (3) governance, organization, workforce, financing, information systems, or equity relevant to sports–medicine integration; (4) implementation or evaluation of physical-activity promotion within healthcare settings; or (5) national policy or professional guidance directly relevant to these domains.

We prioritized peer-reviewed empirical studies, systematic and scoping reviews, and authoritative governmental or professional documents. Records were screened first by title and abstract for relevance to the predefined review scope. Potentially relevant publications were then assessed in full text. Citation chaining and targeted policy searches were used to identify additional sources not captured through PubMed. When several publications described the same policy, program, or institutional arrangement, preference was given to the most recent, authoritative, and directly relevant source. The initial searches identified 11,392 bibliographic records. After removal of duplicates, 10,766 underwent title and abstract screening. One hundred and fifty-eight publications were examined in full text, and 36 peer-reviewed publications were retained for the final synthesis.

### 2.4 Evidence appraisal

Because the review included heterogeneous evidence types, including empirical studies, systematic and scoping reviews, conceptual publications, policy documents, and professional guidance, a single risk-of-bias instrument was not considered appropriate for all sources. Instead, evidence was critically appraised according to source type using a structured set of criteria: methodological rigor, sample size and study design where applicable, directness of relevance to sports–medicine integration, recency, consistency with other evidence, and authority of the issuing organization.

Greater interpretive weight was given to systematic reviews, meta-analyses, large empirical studies, national policy documents, statutory guidance, and publications from recognized professional organizations. Small descriptive studies, single-site implementation reports, and non-comparative program evaluations were treated as contextual or implementation evidence rather than as definitive evidence of effectiveness. Policy and professional documents were used primarily to describe governance structures, service models, or formal recommendations and were not interpreted as evidence of intervention effectiveness unless supported by empirical evaluation.

### 2.5 Data synthesis

Eligible sources were organized thematically according to five implementation domains: (1) governance and institutions; (2) service organization and referral pathways; (3) workforce and professional systems; (4) financing, market participation, and equity; and (5) digital and information integration. Evidence from China was synthesized first to identify current development and implementation constraints. Evidence from the United States, Germany, and Japan was then used comparatively to illustrate alternative mechanisms, strengths, and implementation limitations rather than to rank national health systems. To avoid conflating different levels of evidence, included sources were further classified according to their primary evidentiary function: (1) intervention-effectiveness evidence, including systematic reviews, meta-analyses, and empirical outcome evaluations; (2) implementation evidence, including studies of adoption, referral, fidelity, uptake, and service delivery; and (3) descriptive or normative evidence, including sources addressing governance, policy, professional standards, financing, and health-system organization. Policy documents and professional guidance were therefore used primarily to characterize formal structures, institutional arrangements, and recommendations, and were not interpreted as evidence that sports–medicine integration improves health outcomes. Because of substantial heterogeneity in study design, intervention type, outcome measures, and policy context, quantitative pooling was not undertaken. Findings were synthesized narratively, with the strength and scope of conclusions interpreted in relation to the type and methodological quality of the underlying evidence (Tables 1, 2).

**Table 2:** Comparative characteristics of selected physical activity–health integration models.

**Dimension**	**China**	**United States**	**Germany**	**Japan**
Governance	National policy leadership with separate sports and health administrative systems; growing cross-sector coordination.	Decentralized/pluralist; health systems, professional societies, payers, employers, community and private providers.	Federal/social-insurance model with physicians, statutory insurers, sports organizations and local actors.	National health-promotion framework implemented through ministries, local governments, health and community-care systems.
Typical service mechanism	Community, hospital, fitness-club and multi-actor pilots; exercise prescription developing.	PA assessment, counselling, referral and EIM programs implemented variably across health systems.	Physician “Rezept für Bewegung” recommendations linked to qualified community sport; prevention programs may receive insurance support.	National PA guidance, health check/promotion programs and multidisciplinary community/health services.
Professional system	Developing exercise-prescription standards; credentialing and role boundaries remain uneven.	Relatively mature exercise-professional credentialing (e.g., ACSM-EP/CEP) and allied-health pathways.	Qualified prevention-sport programs and physician/sport-organization collaboration; routine uptake remains limited.	Health exercise instructors and multidisciplinary health-promotion roles supported by national guidance.
Evidence base and limitations	Administrative silos; uneven regional capacity; incomplete reimbursement and credentialing; limited large-scale outcome evaluation.	Fragmented reimbursement and delivery; PA assessment/referral not yet routine; access can depend on payer and local resources.	Low use of exercise prescription in primary care; movement prescription itself is not a routinely reimbursed medical service.	Implementation depends on local workforce and coordination; population ageing creates sustained service and workforce pressure.

## 3 From fragmented health approaches to a holistic health perspective

### 3.1 The value and limits of specialization and treatment-centered care

Modern biomedicine, specialization, evidence-based medicine, and precision approaches have produced major advances in diagnosis, treatment, survival, and disease-specific management. These achievements should not be conflated with the organizational problem of fragmentation. The central limitation is not that disease-focused medicine is intrinsically reductionist or harmful, but that clinical treatment alone cannot address the full set of behavioral, social, environmental, and commercial determinants that shape chronic disease risk and long-term functional health. For conditions strongly influenced by physical inactivity, diet, smoking, sleep, psychosocial stress, and social context, high-quality clinical care and population prevention are complementary rather than competing strategies.

This distinction is important for sports–medicine integration. The objective should not be to replace medicine with exercise or to frame exercise as a universal substitute for clinical treatment. Instead, physical activity can be incorporated into prevention, treatment support, and rehabilitation when the evidence, clinical context, and patient preferences justify it. The policy challenge is to create pathways that connect people to appropriate levels of physical-activity support while preserving medical oversight for those at higher risk and avoiding unnecessary clinicalization of low-risk everyday movement.

### 3.2 Healthism, individual responsibility, and structural determinants

Crawford’s original account of “healthism” described a tendency to locate health problems and their solutions primarily at the level of individual behavior, potentially obscuring the political and social conditions that shape health opportunities (10). This critique remains useful, but it should not be interpreted as a rejection of individual agency. Behavioral counselling, exercise prescription, self-management, and personal responsibility can contribute to health improvement; the problem arises when these are treated as sufficient explanations for population-level disparities.

Physical activity is strongly conditioned by access to safe environments, time, income, transport, disability support, social norms, healthcare access, and the availability of affordable programs. WHO’s physical-activity strategy therefore adopts a whole-of-system approach and explicitly emphasizes equitable access to supportive environments and opportunities (6). This perspective helps avoid two opposing simplifications: attributing health entirely to individual choice, or assuming that public policy alone can generate sustained behavior change without individual engagement. Effective health promotion requires both supportive structures and meaningful personal agency.

### 3.3 Physical activity within a holistic health framework

A holistic health perspective is compatible with, rather than opposed to, rigorous biomedical evidence. WHO conceptualizes health as extending beyond the absence of disease, and contemporary physical-activity guidance recognizes benefits across cardiovascular, metabolic, musculoskeletal, cognitive, and mental-health outcomes (4). For integrated care, health systems should identify physical inactivity and provide brief advice when appropriate. Patients requiring additional support should be referred to qualified exercise or community services. People with chronic conditions or higher clinical risk may also require continued monitoring.

Evidence from primary care indicates that health-professional-delivered or prompted physical-activity interventions can increase physical activity, although effects are generally modest and vary by intervention design and measurement method. A 2022 systematic review and meta-analysis found an average increase of 14 min per week of moderate-to-vigorous physical activity and higher odds of meeting activity guidelines among intervention participants, but substantial heterogeneity remained across trials (11). Other reviews similarly suggest that counselling alone may have limited effects and that continued support, individualized content, referral pathways, and follow-up can be important (12, 13). Thus, the evidence supports integration of physical-activity assessment, counselling, and referral into healthcare in selected contexts, but it should not be interpreted as demonstrating the effectiveness of sports–medicine integration as a comprehensive health-system model. The available literature therefore provides a rationale for testing integrated service pathways rather than evidence that system-wide integration is already effective.

## 4 Sports–medicine integration in China: current development, challenges, and international lessons

In this review, sports–medicine integration is used as an umbrella term for structured coordination among healthcare services, exercise professionals, community and sport organizations, financing mechanisms, and information systems to support physical activity for prevention, chronic disease management, and rehabilitation. To avoid ambiguity between “integration” and “fusion,” the term integration is used consistently throughout this review. Integration can vary in depth-from information sharing and referral to co-located multidisciplinary services-but should be evaluated by whether it improves access, quality, safety, outcomes, and equity. The evidence reviewed in this section should be interpreted at different levels. Studies of physical-activity counselling, exercise referral, or rehabilitation provide evidence regarding specific interventions, whereas national policies, professional standards, and organizational models primarily describe how integration is structured or implemented. The latter should not be interpreted as causal evidence that integration improves population health outcomes. Accordingly, the international comparisons below are used to identify organizational mechanisms, implementation barriers, and potentially transferable design principles rather than to compare the effectiveness of national systems.

### 4.1 Governance and institutional integration

China has strong national policy support for health promotion and national fitness. Healthy China 2030 and subsequent national health planning documents provide a broad mandate to shift attention toward prevention, life-course health, and multisectoral action (7, 14). The Basic Medical and Health Care and Health Promotion Law further clarifies governmental responsibility for health promotion and national fitness while assigning health-related responsibilities across departments (8). These features can facilitate agenda setting and coordinated national priorities.

Policy endorsement, however, does not automatically produce operational integration. A qualitative analysis of Chinese sports–medicine integration policies similarly identified weaknesses in the balance and operational use of policy instruments, indicating a gap between strategic policy support and implementation (15). Sports and health authorities retain distinct administrative mandates, budgets, professional systems, and performance indicators. Evidence from Chinese pilot programs indicates that local implementation models remain heterogeneous and that there is no nationally standardized pathway linking clinical assessment, exercise prescription, community delivery, follow-up, and reimbursement (9). The concentration of early models in cities such as Suzhou and Shanghai also raises questions about transferability to less-resourced regions. More specific policy instruments are therefore needed, including payment rules, service-pricing standards, referral protocols, workforce requirements, and shared performance indicators. And recent empirical work also suggests that public sports services and public health services can produce complementary health benefits, but such findings do not eliminate the need to address institutional coordination, local capacity, and unequal service availability (16).

International comparisons show that decentralized arrangements also require coordination. In Germany, physical-activity promotion involves federal and state actors, physicians, statutory health insurance, sports organizations, and local programs. The “Rezept für Bewegung” initiative links physician recommendations with qualified community sport opportunities. Certified prevention programs can also interact with insurance-based prevention mechanisms. However, the movement prescription remains a recommendation rather than a routinely reimbursed medical prescription, and uptake in primary care has historically been low (17, 18). Germany therefore illustrates both the innovation potential of social-insurance and civil-society partnerships and the implementation challenge of embedding them in routine practice.

The United States provides a different example. Professional bodies such as the American College of Sports Medicine (ACSM) have developed detailed exercise-professional credentials and the Exercise is Medicine initiative (19, 20), while health systems can implement physical-activity assessment, referral, and digital documentation locally. This pluralism can support innovation, but national reviews continue to describe physical-activity assessment, prescription, and referral as far from standard practice and identify reimbursement, interoperability, and system fragmentation as major barriers (20, 21). These contrasts suggest that China should not simply import a foreign model; rather, it should combine national coordination with mechanisms that allow professional specialization, local adaptation, and accountable implementation. A coordinated multi-stakeholder governance structure for China is illustrated in Figure 1.

**Figure 1:**
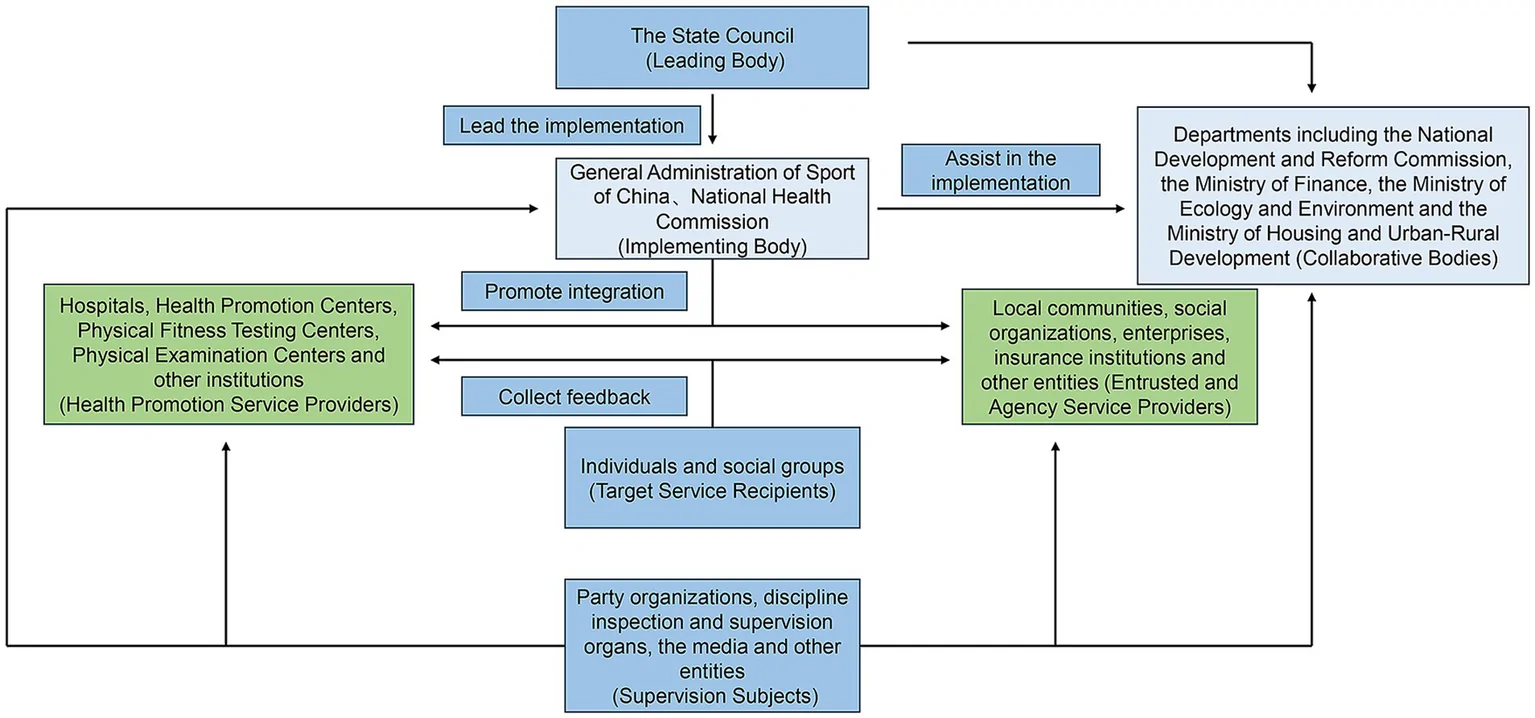
Multi-sector governance and accountability framework for sports–medicine integration in China. The framework is conceptual and is intended to illustrate potential organizational relationships rather than a validated causal model of effectiveness. Blue boxes indicate governmental, coordinating, supervisory, and target-recipient roles, while green boxes indicate direct or commissioned health-promotion service providers. Arrows represent the main pathways of implementation, coordination, service delivery, feedback, and supervision.

### 4.2 Service and organizational integration

Service integration concerns where patients enter the system, who assesses them, how risk is stratified, who delivers exercise support, and how information returns to the referring clinician. In China, existing models include community-based services, hospital-led programs, fitness-club partnerships, and multi-actor collaborations (9). These initiatives demonstrate feasibility but remain uneven in organization and scope. A scalable national approach requires a common service logic rather than a single institutional template. However, routine clinical delivery remains limited. In a Shenzhen study, only 19.2% of patients with chronic diseases reported receiving physical-activity advice from physicians, and comprehensive advice covering frequency, intensity, duration, and type was uncommon (22).

A practical model should distinguish low-, moderate-, and high-risk pathways. Low-risk individuals may need brief advice, community programs, or digitally supported self-management rather than medical supervision. People with stable chronic disease may benefit from referral to qualified exercise professionals or rehabilitation programs with defined communication back to primary care. Higher-risk patients may require clinical exercise testing, specialist assessment, or supervised rehabilitation. This risk-stratified approach can preserve safety without unnecessarily converting ordinary physical activity into a medical procedure.

The international evidence also argues against assuming that referral alone is sufficient. Exercise-referral schemes can improve physical activity and selected cardiometabolic or mental-health outcomes, but effect sizes are often small to moderate and depend on uptake, adherence, program quality, and follow-up. Recent synthesis indicates that newer referral schemes show more favorable results than earlier evaluations, while cost-effectiveness remains variable across settings (12, 23–26). A systematic review of intervention components found that person-centered approaches, screening, and brief advice were associated with better adherence, emphasizing that service quality matters as much as the act of referral itself (27).

For China, the organizational priority is therefore to define a minimum service pathway that can operate across diverse local settings. Core elements should include standardized physical-activity and risk assessment, clear referral criteria, qualified provider directories, follow-up and feedback mechanisms, and outcome measures that can be used for quality improvement. However, the coordinating role of primary care depends on local workforce and service capacity. Additional training, referral resources, and manageable workloads are needed if physical-activity assessment and follow-up are incorporated into routine primary care (28). Community health centers are likely to be important coordination points, but local models should allow collaboration with hospitals, rehabilitation services, sports organizations, and appropriately regulated private providers. Such vertical and horizontal organizational linkages are illustrated in Figure 2.

**Figure 2:**
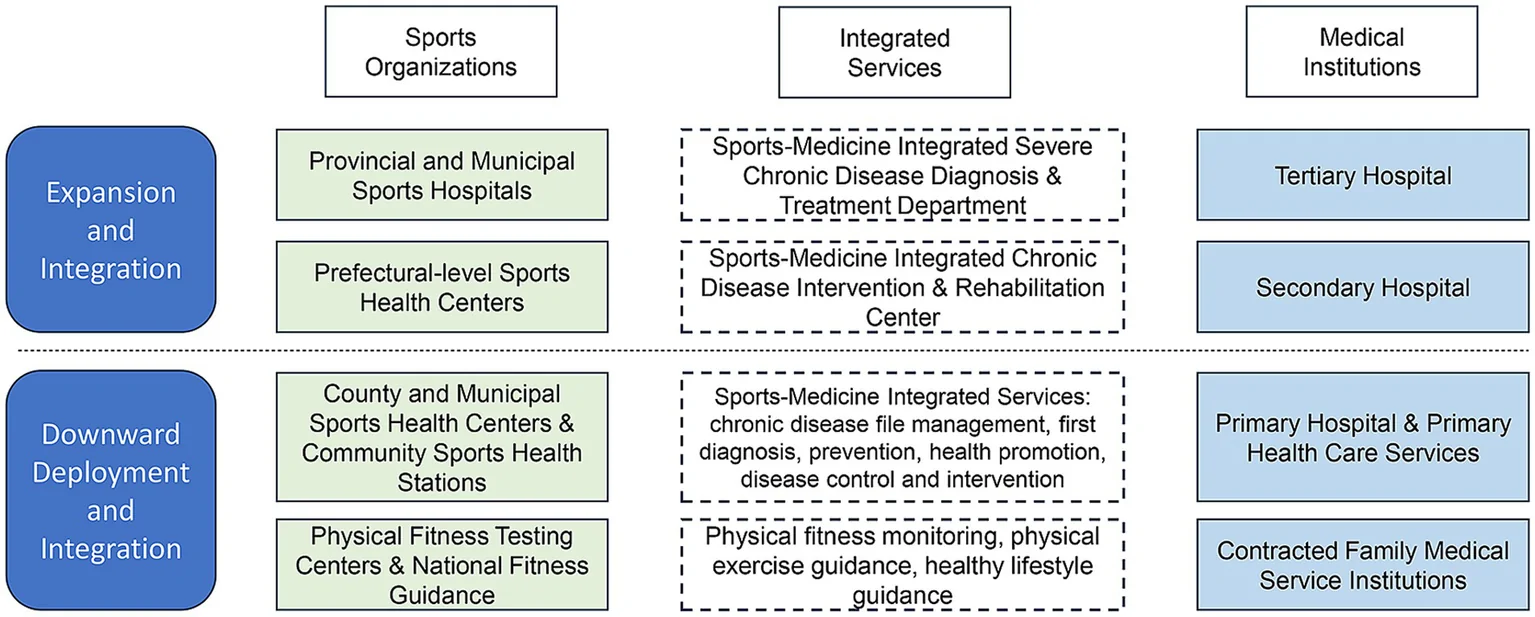
Organizational framework for the integration of sports and medical institutions in China. Green boxes represent sports-sector institutions, light-blue boxes represent medical institutions, and dashed boxes indicate integrated sports–medicine service units or service functions. Dark-blue boxes on the left denote the two major organizational pathways: expansion and integration at higher institutional levels, and downward deployment and integration toward primary and community-based services. The framework illustrates the vertical alignment of sports organizations, integrated services, and medical institutions across tertiary, secondary, and primary levels.

### 4.3 Workforce and professional integration

Workforce capacity is a central bottleneck because sports–medicine integration depends on professionals who can translate clinical information into safe, individualized, and sustainable physical-activity plans. China has recently strengthened technical guidance through the Consensus Statement of Chinese Experts on Exercise Prescription, which distinguishes fitness and medical exercise prescriptions and addresses risk assessment and exercise safety (29). Nevertheless, the broader professional system remains fragmented. Exercise specialists, rehabilitation professionals, physicians, nurses, and public-health practitioners may have overlapping functions but different education pathways, institutional affiliations, and recognized scopes of practice.

The United States illustrates one advantage of a mature professional certification ecosystem. ACSM distinguishes general exercise-physiology and clinical exercise-physiology credentials. The clinical pathway requiring university training and substantial supervised clinical experience, while recent certification changes further emphasize clinical testing, risk management, and multidisciplinary collaboration (30, 31). Such credentialing does not solve access or reimbursement problems, but it creates clearer expectations about competence and referral.

Japan provides another relevant reference point. The Physical Activity Guide for Health Promotion 2023 targets both the general public and relevant professionals. These include health exercise instructors, physicians, public-health nurses, dietitians, policymakers, workplace managers, and health and long-term-care professionals. The current Health Japan 21 framework also links individual behavior with social environments and life-course health (32, 33). This illustrates how workforce development can be embedded within a broader national health-promotion strategy rather than treated solely as a new academic specialty.

For China, the main priority is not simply to create more training programs. A national workforce strategy should define competency tiers, protected professional titles where appropriate, referral boundaries, continuing education requirements, and accountability for clinical risk. It should also address rural and primary-care workforce shortages. Over-specialization could increase cost and reduce accessibility, so low-risk physical-activity promotion should remain deliverable by appropriately trained generalist health workers and community providers, while complex cases are escalated to specialist exercise or rehabilitation professionals.

### 4.4 Financing, market participation, and equity

Market size is not an adequate proxy for the public-health value of sports–medicine integration. The more important financing questions are who pays for assessment, counselling, exercise referral, supervised programs, and follow-up; which services are eligible for insurance or public funding; and whether financing arrangements widen or reduce inequity. Evidence from exercise-referral schemes suggests that such programs can be cost-effective in some settings, but estimates are sensitive to assumptions about effectiveness, uptake, and program cost (26). Economic evaluation should therefore accompany scale-up rather than be inferred from industry growth.

Germany illustrates a model in which statutory health insurance supports qualified prevention activities and physicians can recommend community exercise. By contrast, the United States relies more heavily on mixed payer arrangements involving health systems, employers, professional services, and private or community providers. These arrangements can generate diverse programs and innovation, but they may also create gaps in coverage and continuity. China’s government-led system can potentially use public financing and insurance policy to reduce such fragmentation (34), yet limited reimbursement rules for exercise services and large differences in local fiscal and service capacity remain important constraints (28, 35). It thus indicates that clearer rules are needed to define which exercise-related services can be publicly funded or reimbursed and how assessment, exercise prescription, supervised training, and follow-up should be priced.

Equity must be treated as a core outcome rather than a secondary consideration. Early Chinese sports–medicine pilots have been concentrated in urban areas, and the availability of specialized professionals, fitness facilities, rehabilitation services, and digital platforms differs markedly across regions. Low-income populations, rural residents, older adults, and people with disability may face greater financial, transport, digital, or functional barriers to participation (36, 37). These barriers may be particularly important in rural areas, where specialist personnel, rehabilitation services, and referral options are more limited. Global physical-activity policy explicitly warns that promotion strategies should not exacerbate sex, age, socioeconomic, or geographical inequalities (3, 6). A national integration strategy should therefore monitor who is reached, who completes programs, who benefits, and who bears out-of-pocket costs.

Private-sector participation can complement public capacity, particularly in digital platforms, fitness facilities, rehabilitation technology, and service innovation. The broader public-health functions of sports–medicine integration across prevention, chronic-disease management, and rehabilitation are summarized in Figure 3. However, such integration requires clear quality standards, conflict-of-interest safeguards, data governance, and transparent reimbursement rules to prevent commercialization from driving unnecessary services or widening access gaps. The objective should be population health gain rather than expansion of a “health industry” as an end in itself.

**Figure 3:**
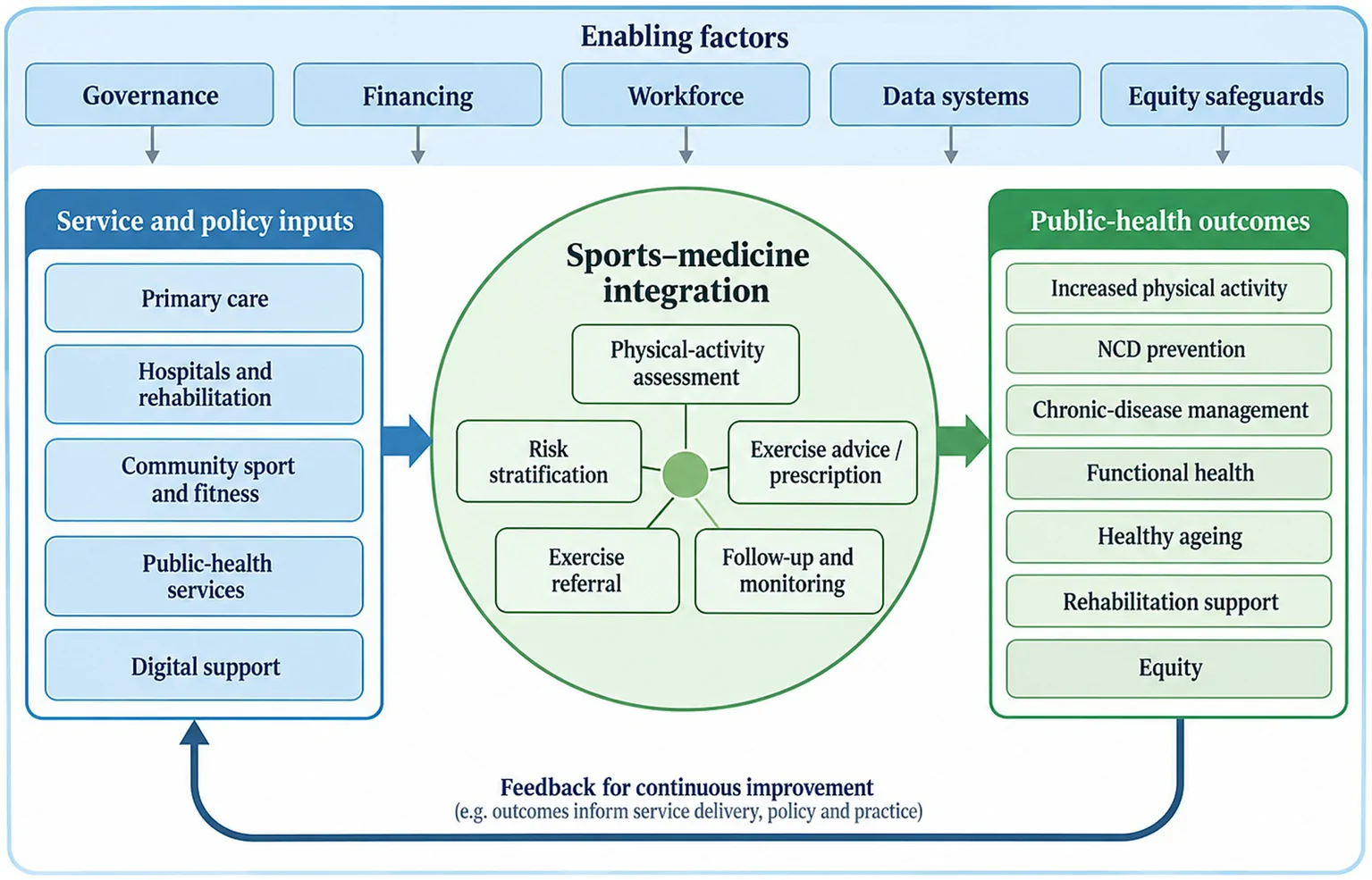
Integrated framework for sports–medicine-related industries in health promotion and chronic disease prevention. Blue elements indicate service inputs and enabling system conditions, whereas green elements indicate core integration functions and public-health outcomes. Arrows represent the pathways linking service delivery, integration, health outcomes, and feedback for continuous improvement.

### 4.5 Digital and information integration

Information integration is essential because referral pathways fail when physical-activity assessment, exercise plans, rehabilitation records, and follow-up outcomes remain in separate systems. A useful digital architecture should enable structured physical-activity assessment, clinical risk information, referral status, program participation, and outcome feedback while applying appropriate privacy and consent safeguards.

Experience from the United States demonstrates both the potential and the challenge of embedding physical activity into routine health information systems. Exercise is Medicine was implemented across five primary-care clinics within a large academic health system. A Physical Activity Vital Sign was incorporated into the electronic health record, demonstrating feasibility, adoption, and maintenance. However, implementation fidelity varied across clinics and program components (38, 39). National policy analyses nevertheless note that standardized measures, interoperability, payer incentives, and referral infrastructure remain incomplete (21).

For China, digital integration should not be limited to creating a new stand-alone platform. A more sustainable approach would develop interoperable data standards connecting existing hospital, primary-care, public-health, national-fitness, and rehabilitation systems. Digital tools could support remote coaching and follow-up, particularly where specialist services are scarce, but they may also widen the digital divide if older, rural, low-income, or disabled populations cannot access or use them (40). Digital services should therefore complement, rather than replace, face-to-face, telephone, and community-based support. Data minimization, cybersecurity, professional accountability, and transparent rules for commercial platform access should therefore be built into system design from the outset.

## 5 Toward an integrated framework for health promotion

### 5.1 Potential benefits and unresolved risks

Sports–medicine integration has a plausible and evidence-informed role in public health, but its benefits should be stated with appropriate precision. Physical-activity promotion in healthcare can increase activity and support selected health outcomes, and integrated referral can help connect patients to resources beyond the clinic (11, 23). The strongest rationale is therefore not that exercise replaces medicine, but that healthcare encounters can identify inactivity, assess risk, initiate advice, and connect people to sustained support.

At the same time, several risks require explicit consideration. First, the evidence base for large-scale sports–medicine integration as a system-level intervention in China remains limited. Most published examples describe local programs, policy models, or specific exercise interventions rather than population-wide causal effects on morbidity, utilization, or healthcare costs. Evidence concerning long-term effectiveness, cost-effectiveness, scalability, healthcare utilization, regional transferability, and distributional effects remains limited. Accordingly, the framework proposed in this review should be interpreted as an evidence-informed model for implementation and evaluation, rather than as a model whose effectiveness has already been established. Second, expanding clinical control over everyday physical activity could medicalize behavior that is better supported through schools, workplaces, transport policy, parks, sport, and community environments. Third, unequal access to specialists, facilities, insurance coverage, and digital services could widen existing health inequalities. Fourth, new professional and institutional boundaries may create duplication, referral delays, or unnecessary cost if roles are not clearly defined.

These concerns do not argue against integration; they define the conditions under which integration should proceed. A proportionate model should reserve clinical intensity for people who need it, while making community physical activity easy, affordable, and non-medical for the majority of the population.

### 5.2 Priorities for implementation in China

First, governance should move from parallel policy statements toward joint operational standards. National health and sports authorities should define shared objectives, referral pathways, minimum service requirements, outcome indicators, and accountability mechanisms, while allowing local adaptation. Pilot programs should be evaluated against common metrics before broad replication.

Second, a tiered service model should be established. Routine physical-activity assessment can be incorporated into primary care where feasible, but referral intensity should be based on clinical risk, functional limitation, and patient need (22, 28). Community sport and active-living programs should remain the default option for low-risk populations, with qualified exercise professionals and clinical rehabilitation reserved for more complex cases.

Third, workforce development should focus on competencies and recognized scopes of practice. National standards should distinguish health education and basic physical-activity counselling from exercise programming for chronic conditions and from clinical exercise testing or rehabilitation. Credentialing systems should be linked to education quality, supervised experience, continuing professional development, and referral eligibility.

Fourth, financing and service-pricing mechanisms should be tested together. Demonstration projects should clarify which services are eligible for public funding or insurance reimbursement, how services should be priced, and whether these arrangements reduce out-of-pocket costs and urban–rural inequalities (28, 35).

Fifth, information systems should support rather than duplicate existing health infrastructure. Standardized data fields for physical activity, referral, participation, and outcomes could enable continuity of care and population monitoring. Interoperability, privacy, cybersecurity, and digital inclusion should be explicit design criteria (40). Non-digital alternatives should remain available for populations with limited digital access or literacy.

Finally, implementation should be accompanied by a stronger research agenda. Priorities include pragmatic trials in real-world health systems, longitudinal evaluation of chronic-disease outcomes, implementation-science studies of adoption and sustainability, comparative studies across urban and rural settings, health-economic evaluation, and equity-focused analyses. These forms of evidence are necessary to determine which models work, for whom, at what cost, and under what local conditions.

## 6 Conclusion and future directions

Physical activity should play a more systematic role in health promotion, chronic-disease prevention, treatment support, and rehabilitation, but effective sports–medicine integration requires more than policy endorsement. China has a strong policy foundation for linking national fitness and health, yet implementation remains constrained by institutional fragmentation, uneven local capacity, workforce and credentialing gaps, uncertain financing, and incomplete information integration. International experience further indicates that no single institutional model is universally transferable; effective integration depends on clear responsibilities, professional competence, sustainable financing, accessible community services, interoperable information systems, and continuous evaluation.

Future development in China should therefore focus on testing which models are effective, scalable, equitable, and cost-effective rather than assuming that deeper integration is inherently beneficial. A risk-stratified and person-centered approach represents one plausible model for future implementation, supporting routine physical activity through community-based services while directing higher-risk populations to qualified clinical and exercise professionals. Its effectiveness, scalability, cost-effectiveness, and equity should, however, be established through prospective evaluation rather than assumed from existing intervention-level evidence.
